# Interactions of the CSF3R polymorphism and early stress on future orientation: evidence for the differential model of stress-related growth

**DOI:** 10.1017/S2045796024000581

**Published:** 2024-10-03

**Authors:** Yiqun Gan, Lizhong Wang, Yidi Chen, Lei Zheng, Xiaoli Wu, Gang Chen, Yueqin Hu

**Affiliations:** 1 School of Psychological and Cognitive Sciences and Beijing Key Laboratory of Behavior and Mental Health, Peking University, Beijing, China; 2 Hunan Provincial Key Lab on Bioinformatics, School of Computer Science and Engineering, Central South University, Changsha, P. R. China; 3 WeGene, Shenzhen Zaozhidao Technology Co. Ltd., TianAn CyberTech Plaza I, Shenzhen, P. R. China; 4 School of Economics and Management, Fuzhou University, Fuzhou, China; 5 Shenzhen WeGene Clinical Laboratory, Haikexing Industrial Park, Shenzhen, P. R. China; 6 School of Psychology, Beijing Normal University, Beijing, China

**Keywords:** CSF3R, future orientation, gene × environment interaction, genome-wide interaction studies, threshold model of stress-related growth

## Abstract

**Aims:**

This study aims to explore the concept of future orientation, which encompasses individuals’ thoughts about the future, goal-setting, planning, response to challenges and behavioural adjustments in evolving situations. Often viewed as a psychological resource, future orientation is believed to be developed from psychological resilience. The study investigates the curvilinear relationship between childhood maltreatment and future orientation while examining the moderating effects of genotype.

**Methods:**

A total of 14,675 Chinese adults self-reported their experiences of childhood maltreatment and their future orientation. The influence of genetic polymorphism was evaluated through genome-wide interaction studies (GWIS; genome-wide association study [GWAS] using gene × environment interaction) and a candidate genes approach.

**Results:**

Both GWAS and candidate genes analyses consistently indicated that rs4498771 and its linked single-nucleotide polymorphisms, located in the intergenic area surrounding CSF3R, significantly interacted with early trauma to influence future orientation. Nonlinear regression analyses identified a quadratic or cubic association between future orientation and childhood maltreatment across some genotypes. Specifically, as levels of childhood maltreatment increased, future orientation declined for all genotypes. However, upon reaching a certain threshold, future orientation exhibited a rebound in individuals with specific genotypes.

**Conclusions:**

The findings suggest that individuals with certain genotypes exhibit greater resilience to childhood maltreatment. Based on these results, we propose a new threshold model of stress-related growth.

## Introduction

The phrase ‘what does not kill us makes us stronger’ suggests that the role of stress exposure should be reconsidered, as it profoundly influences our understanding of mental health (Seery *et al.*, 2010). The stress inoculation theory (Kim-Cohen and Gold, 2009; Southwick and Charney, 2012) provides a theoretical framework for understanding the relationship between stress exposure levels and stress-related growth, in which exposure to particular levels of stress leads to an enhanced capacity for coping with future obstacles. However, the findings of studies on this topic have been inconsistent. Stress-related growth following exposure to stress does not always occur. This inconsistency could be owing to different interactions between individual and environmental characteristics.

**Figure 1.**
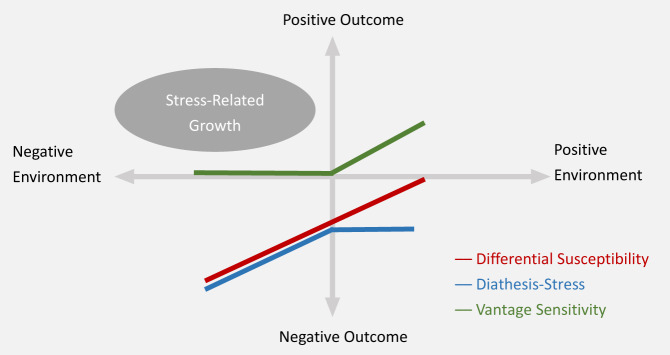
Illustration of the different models describing person × environment interaction.

*Note*: The coloured models are existing theories whereas stress-related growth is proposed in this article.

Three existing theories, summarized in Fig. 1 (Jolicoeur-Martineau *et al.*, 2020; Rioux *et al.*, 2016), explain the interactions between person and environment: the diathesis–stress model, the differential susceptibility model and the vantage sensitivity hypothesis (Belsky and Pluess, 2009). The diathesis–stress model (Monroe and Simons, 1991) proposed that the interaction between a predispositional vulnerability and stress would lead to a psychological disorder. The differential susceptibility model (belsky and Pluess, 2009) then extended the classic diathesis–stress theory to describe processes in a positive environment and describes a group of individuals that are sensitive to negative exposures but also susceptible to positive exposures. Both models are primarily focused on psychopathology. Yet, positive adaptation is conceptually different from the absence of psychopathology. Later, Pluess and Belsky (2013) introduced the term vantage sensitivity to characterize the ‘positive side’ of differential susceptibility in response to positive experiences. However, contrastive effects (Rioux *et al.*, 2016) such as how individuals could be positively affected by negative environments were not yet theorized. This study attempts to fill this gap by specifically addressing the association between stress exposure and stress-related growth.

### Early adversity and mental health: linear relationship and quadratic hypothesis

The conventional academic view posits a linear relationship between stress and mental health, with stressful events typically increasing the risk of negative mental health outcomes. This concept, widely supported by empirical research, aligns with the dose–response theory that describes a linear relationship between stress events and mental health (Hou *et al.*, 2020; Sala *et al.*, 2014; Turner and Lloyd, 1995). Childhood stressors often have more enduring effects than those in adulthood (Zannas and West, 2014), and numerous studies verify the negative psychological consequences of adverse distant events (Lähdepuro *et al.*, 2019).

However, the more recent focus on positive psychology reveals that stress can also result in positive changes like enhanced social resources, personal development and improved coping skills (Ord *et al.*, 2020). Moderate stress exposure may even be beneficial for obtaining psychological resources (Lyons *et al.*, 2010a), leading researchers to question the linear relationship and propose a potential quadratic relationship between stress and mental health (Seery *et al.*, 2010).

Subsequent research has largely supported this quadratic hypothesis, demonstrating it across various populations and situations (Holtge *et al.*, 2018). For instance, moderate lifelong stress is related to psychological resilience in breast cancer survivors (Dooley *et al.*, 2017), and distant stress has long-term effects on mental health in older adults (Mclafferty *et al.*, 2018). Childhood stress, previously viewed as entirely negative, is now recognized for its potential positive outcomes (Finch and obradović, 2017; Höltge *et al.*, 2019).

Two reasons for these recent findings have been identified. First, some studies only selected childhood trauma samples, leading to a skewed representation of stress exposure (Lemoult *et al.*, 2020; Steine *et al.*, 2017). Second, in the verification method of the quadratic hypothesis, a multivariate linear regression method is generally adopted, wherein the first-order term is added to the model first, followed by the simultaneous addition of the first-order and second-order terms (Seery and Quinton, 2016). This means that the quadratic relationship places constraints on the linear relationship, and the linear relationship can only be supported if the quadratic relationship is not supported (Katz *et al.*, 2019).

Our recent meta-analysis of 24 studies involving 27,547 participants, collecting 5,036 cross-sectional samples and tracking 1,173 participants over a year, found that proximal stress events aligns with the linear hypothesis, while the stress events distal stress events fits the quadratic hypothesis (Ma & Gan, under review). This indicates that the occurrence time of stress moderates the relationship between stress and well-being. The growth process from stress events requires active cognitive components like future-oriented thinking, which take considerable time to take effect (Brooks *et al.*, 2017; Crane *et al.*, 2019).

### Post-traumatic growth, stress-related growth and future orientation

Post-traumatic growth (PTG) theory posits that traumatic events can shatter an individual’s cognitive schema, necessitating its reconstruction (Tedeschi and Calhoun, 2004; Tedeschi and Lg, 1996). This process can lead to a greater appreciation of life, improved personal relationships, an increased sense of personal strength, recognition of new possibilities in life and spiritual development. The extent and nature of PTG can vary greatly among individuals and depends on the characteristics of the trauma, the individual and the recovery environment (Tedeschi *et al.*, 2018). Joseph and Linley (2006) describe PTG as an active and ongoing process, positing that PTG is not a direct result of trauma but arises from the struggle with new reality in the aftermath of trauma. Moreover, Janoff-Bulman (1992) shattered assumptions theory also offers a crucial perspective on how traumatic experiences can profoundly shake one’s fundamental beliefs about the world, potentially paving the way for growth. More recent research findings have also linked neurobiological factors to stress-related growth. For example, hippocampal volume is associated with the degree of growth following trauma (Rubin *et al.*, 2016).

Stress-related growth, a similar concept, refers to the perception or experience of improvement or positive change due to the struggle with a challenging life circumstance or major life crisis (Park and Ai, 2006). This growth is often attributed to the development of coping strategies, social support and cognitive processes such as positive reappraisal (Carver and Antoni, 2004). Casellas‐Grau *et al.* (2017) incorporate current perspectives on the topic and systematically reviewed stress-related growth among women with breast cancer.

However, both stress-related growth and PTG are complex processes that are not experienced by everyone who encounters stress (Zoellner and Maercker, 2006). Not all changes following trauma are positive, and the perception of growth does not necessarily equate to a reduction in distress or improved mental health (Bonanno *et al.*, 2011). Thus, a research gap exists concerning the mechanisms underlying stress-related growth, and there is a need to identify factors that may explain these individual differences. This knowledge is crucial to develop interventions that foster growth and resilience following stressful events.

Although it is well established that early trauma may result in psychopathology (Mclaughlin *et al.*, 2020), a moderate level of exposure to stress may lead to better mental health and well-being (Ashokan *et al.*, 2016). These results were explained considering the ‘‘stress inoculation model,’ which posits that the effects of early life stress on mental health can be represented by an inverted U-shaped curve, in which extremely high or low levels of early life stress causes poor coping capacity, while a moderate level of early life stress is optimal in preparing individuals for coping with future stress (Lyons *et al.*, 2010b). In an influential study, Seery *et al.* (2010) reported the findings obtained based on an inverted U-shaped curve and demonstrated that exposure to moderate stress helps promote proactive coping and thereby increases resilience. These findings suggest that the role of stress exposure should be reconsidered, as it could profoundly affect our understanding of mental health.

We found that the occurrence time of stress moderates the relationship between stress and well-being; specifically, proximal stress events align with the linear hypothesis, while distal stress events fit the quadratic hypothesis (Ma & Gan, under review). The growth process from stress events requires active cognitive components like future-oriented thinking, such as planning, anticipation and goal-setting, which take considerable time to take effect (Brooks *et al.*, 2017; Crane *et al.*, 2019). These cognitive components are inherently linked to the process of growth following stress, thereby justifying its use as a proxy measure.

Future orientation is a process involving the dynamic development of mental resources despite adversity. Future orientation includes not only information about how individuals think about the future but also how they set goals, plan for the future, respond to challenges and adjust their behaviour and reassess their goals as situations evolve. Individuals with a high future orientation tend to have a long-term perspective and can prepare in advance for the possibility of stress, plan for the future and evaluate their achievement and accordingly revise their plan to match their goal timeline. Contrastingly, individuals with low future orientation tend to be overwhelmed and have difficulty in generating effective coping strategies when facing stress (Nurmi and Pulliainen, 1991). Future orientation is often regarded as a psychological resource that can develop from psychological resilience (Seginer, 2008). For example, Seginer (2008) proposed a model in which psychological resilience leads to future orientation, with a series of intrapersonal and interpersonal traits serving as moderators. Further, the findings of Cui *et al.* (2020) highlighted the role of future orientation in the development of resilience among maltreated youth. Taken together, future orientation should be a reasonable indicator of stress-related growth.

### Individual differences and gene: moderators for the adversity–resilience relationship

Owing to individual differences in stress sensitivity, ‘moderate level’ is a vague term. Although there is evidence indicating that the relationship between stress levels and coping capacity can be depicted by an inverted U-shaped curve, previous research has shown that only a proportion of individuals learned to enhance their coping capacity, which resulted in an increase in resilience, despite exposure to the same levels of stressful events (Cicchetti *et al.*, 2012). This suggests that exposure to a level of stress that is beneficial to one individual might be harmful to another. One explanation for this is that certain individuals have previously experienced stress, and thus developed coping strategies, owing to which they experienced stress-related growth and developed greater resilience. Future-oriented coping is a strategy for coping with stress in advance. This line of research indicates the importance of individual differences in understanding the relationship between a specific stress level and stress-related growth.

Individual-level variables have been thought to play a role in the association between stress exposure and the development of resilience (Feder *et al.*, 2009; Rutten *et al.*, 2013). However, the role of genetic differences has unfortunately been greatly overlooked to date (Cicchetti *et al.*, 2012; Kim-Cohen and Gold, 2009; Rutten *et al.*, 2013). Genetic composition has been widely regarded as an individual-level variable and its importance has been demonstrated in the modulation of psychopathological variables and positive psychological health (Cicchetti *et al.*, 2012; Massat *et al.*, 2004). Further, our prior study found that genes could interact with stress to develop psychological resources in different stressful situations (Gan *et al.*, 2019).

Individuals appear to exhibit a different capacity for resisting a specific stressor, resulting in different levels of growth. Previous genetic studies have shown that not every individual could achieve mental growth after experiencing early life adversities (Cicchetti *et al.*, 2012). The interaction between early childhood maltreatment and genetic predispositions in shaping future orientation is a complex and nuanced issue. Genetic factors can influence how individuals respond to environmental stressors, including maltreatment (Caspi *et al.*, 2002). Studies on other genetic polymorphisms, such as those of the serotonin transporter gene (*5-HTT*), have shown that individuals with certain variants of this gene who are exposed to stressful life events have an increased risk of developing depression (Caspi *et al.*, 2003). Similarly, certain polymorphisms of the *FKBP5* gene are associated with an increased risk of post-traumatic stress disorder following exposure to severe trauma (Binder *et al.*, 2008). Cornelis *et al.* (2010) review and future directions on gene–environment interactions and post-traumatic stress disorder also expand on this aspect.

Prior studies have reviewed several polymorphisms that are associated with resilient adaptation, such as hypothalamus–pituitary–adrenal (HPA) axis-related genes (e.g., *CRHR1, FKBP5*), serotonin transporter genes (e.g., *5-HTTLPR*), *COMT, NPY* and *BDNF* (Feder *et al.*, 2009), and suggested that genes could be a critical factor in individual differences in such adaptation. Inspired by animal models, researchers have noted a possible association between immune cells and resilience to stress (Tsyglakova *et al.*, 2019). However, there is a paucity of research on genes regulating inflammatory and immune responses as indicators of individual differences and their involvement in the pathway from the stressful experience to the development of future orientation.

### GWAS using gene × environment interaction (GWIS) and candidate gene approaches

The candidate gene approach was widely used in the early studies of gene × environment interaction (G × E) interactions. The basic principle of candidate gene analysis is based on prior biological or functional knowledge, which tests the hypothesis that selected genes interact with factors to shape a complex trait. However, there are some debates about candidate gene studies. First, these studies could have a greater number of potential statistical tests that increase the occurrence of false positives when testing for interaction effects, making the studies less reproducible (Munafò *et al.*, 2009). Second, candidate gene studies were subject to publication bias. This suggests that high-quality replicated studies with interaction validation findings are needed to improve the reproducibility of the field (Duncan and Keller, 2011). In genetic–psychological trait association studies, there has been a shift from hypothesis-driven studies examining a small number of candidate genes to more agnostic (hypothesis-free) genome-wide screening for polymorphisms contributing to complex psychological traits. By modelling the interaction between single-nucleotide polymorphism (SNP) alleles and early stress status on the Childhood Trauma Questionnaire (CTQ-R) score through genome-wide interaction studies (GWIS), we sought to identify potential candidate genes that have passed the preliminary screening. Moreover, the reliability of this study was enhanced by cross-validation.

### The present study

Based on mental resilience and stress inoculation theory, we believe that individuals can grow mentally after exposure to stressful events; however, this growth is conditioned on specific environmental and individual characteristics. Some specific range of stimulus intensities and specific genotypes may enable or facilitate this growth.

The negative environmental exposure or adversity used in this study is childhood trauma – a commonly used environmental variable for the study of G × E interaction (Taylor, 2010). To identify the related genes, this study adopted a hybrid method that combines the candidate approach and the GWAS approach. First, the GWAS approach was used to screen for the potential genes relevant to the interaction of gene and childhood maltreatment on future orientation. Next, *CSF3R* was used as the focus candidate gene owing to the preliminary results in GWAS and its impact in the stress and coping process (Kawai *et al.*, 2007; Le-Niculescu *et al.*, 2011).

Based on the above rationale, we hypothesized a quadratic association (an inverted U-curve) between childhood maltreatment and future orientation, suggesting a middle level of childhood maltreatment as optimal. Linear and polynomial models up to the fourth power were examined as competing hypotheses. We also hypothesized an interaction effect between the CSF3R and childhood maltreatment. More specifically, future orientation would be less impaired or stimulated by childhood maltreatment among individuals carrying certain alleles. A large nationwide sample of Chinese adults was used to examine the above hypotheses.

## Methods

### Participants

We recruited 14,675 adult participants drawn from the customer base of WeGene, a personal genetic company, who had been genotyped by ∼700,000 SNPs on microarrays (38% on Affymetrix WeGene V1 array and 62% on Illumina Infinium Global Screening Array-24 v2.0 BeadChip array) as part of the WeGene Personal Genome Services (Kang *et al.*, 2020). WeGene operates its own user community and offers various research opportunities through its online user community forum. By participating in research, users can earn points redeemable for virtual goods, physical rewards or donations to other users – with 200 points generally equivalent to one Chinese yuan (RMB). Participants provided informed consent online and participated in the research online through organic posts on WeGene online platform. The customers of WeGene cover people over 18-years-old and are representative in terms of age, region and occupation. After excluding 143 people who failed the validity items test, the final sample contained 14,675 people. The validity test comprised five pairs of questions, with questions in each pair having the same meaning but placed at different locations in the questionnaire. Participants were considered to have failed the validity test if they gave opposite responses to any of the paired questions. Each participant was rewarded 500 bonus points in their WeGene account upon completion of this study, regardless of whether they passed the validity test.

All procedures contributing to this work comply with the ethical standards of the relevant national and institutional committees on human experimentation and with the Helsinki Declaration of 1975, as revised in 2008. All procedures were approved by the Committee for Protecting Human and Animal Subjects School of Psychological and Cognitive Sciences, Peking University (no. I2018-10-03e).

All participants were of Chinese Han ethnicity. Their mean age was 28.17 years (SD: 7.08; range: 18–65 years). There were 6,363 (43.4%) men and 8,267 (56.3%) women, while 45 (0.3%) had missing data. Among participants, 3,601 (24.5%) had a master’s degree or higher, 8,614 (58.7%) had a bachelor’s degree, 1,597 (10.9%) were at junior college level, and the rest (5.9%) had a high school diploma. Most were either employed (84.5%) or full-time university students (15.5%).

### Materials

#### Revised version of CTQ-R

The CTQ-R is a self-report instrument used to retrospectively assess the frequency and severity of different types of childhood maltreatment (Bernstein *et al.*, 1997). The Cronbach’s alpha coefficient was 0.97 in Bernstein’s study, demonstrating excellent reliability. The CTQ-R contains 14 items adapted from the CTQ Short Form. We did not include items related to sexual abuse owing to Chinese cultural taboos around sex. All items were rated on a five-point Likert-type scale with anchors of ‘never’ (1) and ‘very often’ (5); thus, higher scores indicated more abuse. The scale demonstrated a good unidimensional structure, with a Cronbach’s alpha coefficient of 0.890 for the current sample.

#### Future orientation inventory

A future-oriented coping inventory was used in GWAS, which is a measure of future orientation and assesses the degree that individuals are likely to opt and prepare themselves for future challenges and stress (Gan *et al.*, 2007). A sample item is, ‘Before disaster strikes, I am well-prepared for its consequences’. It is a reliable and valid measure for future orientation, and many studies have used this instrument to measure individuals’ future orientation (Prochniak and Prochniak, 2021; Serrano *et al.*, 2021). Participants responded to each item using a five-point Likert-type scale that ranged from ‘very untrue for me’ (1) to ‘very true for me’ (5). The 16-item scale demonstrated good psychometric properties, with a Cronbach’s alpha coefficient of 0.873 for the current sample.

### Genome-wide association study

A GWAS analysis was performed for the interaction effect of the CTQ-R and SNP allele on future orientation (dependent variable) in the WeGene cohort using PLINK 1.9. Since GWAS analysis requires binary input, the continuous variable CTQ-R was dichotomized, with the lower 27% (CTQ-R ≤ 13) as controls and the upper 27% (CTQ-R ≥ 23) as cases. The middle 46% were excluded, leading to a final GWAS sample size of 6,504. This case–control approach is essential in GWAS and is often used in psychopathology to detect loci that contribute to a specific trait. PLINK command ‘–gxe’ estimates the difference in allelic association with a quantitative trait (future orientation) between two groups (CTQ-R cases vs. controls) producing effect estimates on each group and a test of significance for the interaction between the SNP allele and future orientation status. The interaction *P*-value reflects the difference between the regression coefficient of the allelic effect in a linear model for the future orientation scale in CTQ-R cases and the same regression coefficient in a linear model for future orientation in CTQ-R controls. The future orientation interaction effect was defined as the difference in allele effect between CTQ-R case and control groups. We used the *Z*-transformed future orientation score to fit the normal distribution.

### Genotyping, quality control and imputation

The cohorts were genotyped following WeGene’s protocols. Individuals were excluded from further analysis based on sex mismatches, disproportionate levels of individual missingness (>5%), evidence of relatedness (removing one from each pair within the second-degree relationship identified by relationship inference software KING with parameter ‘–unrelated – degree 2’) (manichaikul *et al.*, 2010), inbreeding coefficient above 0.2 or below 0.2 (known as heterozygosity *F*-statistic) and being non-Han Chinese (assessed by principal component analysis and two-dimensional clustering analysis (bycroft *et al.*, 2018) including the 1000 Genomes Project Phase 3 data). The quality control parameters used for the exclusion of individuals and SNPs were the following: SNP missingness >0.02; and deviation of an SNP from the Hardy–Weinberg equilibrium <1e-5.

Imputation of genotype data was performed using Eagle/Minimac4 with default parameters (with a chunk size of 10 Mb and step size of 3 Mb) against the 1000 Genomes project Phase 3 v5 reference haplotypes. Post-imputation filtering was achieved by removing SNPs with imputation quality (e.g., Minimac R2) less than 0.3, MAF less than 1% or a missing rate of more than 2%.

### Statistical analyses

First, we examined the curvilinear relationship between childhood maltreatment (linear, quadratic, cubic and quartic terms of CTQ-R scores) and future orientation using regression analyses. Second, we performed GWAS on future orientation to identify candidate genes, as described above. Finally, we examined the interaction effect between candidate genes and CTQ-R on future orientation using regression analyses. To ensure rigor and to exclude alternative explanations, population stratification information, such as sex and age, was controlled for as covariates. The sample was randomly split in half and cross-validated where applicable.

## Results

### Descriptive statistics and the curvilinear relationship between childhood maltreatment and future orientation

The mean and SDs of the major variables are presented per CSF3R genotype in Table 1. We first assessed the linear and nonlinear associations between CTQ-R and future orientation. Regression analyses were performed with sex, age and education controlled; and the linear, quadratic, cubic and quartic term of CTQ-R score entered as predictors. The results presented in Table 2 suggested that the cubic model that includes a linear, quadratic and cubic term of CTQ-R best described the nonlinear relationship between CTQ-R and future orientation, with the smallest Akaike information criterion, largest likelihood and highest *R*^2^ among all models. Further, it explained a significant incremental variance compared to the linear model, Δ*R*^2^ = 0.6%, *F*[2,14616] = 45.71, and to the quadratic model, Δ*R*^2^ = 0.1%, *F*[1,14616] = 19.23. The regression coefficients of the linear, quadratic and cubic terms were all significant except for the quartic term, *β*_1_ = −0.20, 95% CI for *B* = [−0.226, −0.175], *t* = 15.29, *P* < .001; *β*_2_ = −0.24, 95% CI for *B* = [0.067, 0.117], *t* = 7.27, *P* < .001; *β*_3_ = −0.12, 95% CI for *B* = [−0.016, −0.006], *t* = 4.39, *P* < .001; and *β*_4_ = 0.02, 95% CI for *B* = [−0.002, 0.003], *t* = 0.24, *P* = .811.

**Table 1.** The curvilinear relationship between future orientation and early adversity

	Linear model		Quadratic model		Cubic model		Quartic model	
	*B*	SE	*B*	SE	*B*	SE	*B*	SE
Gender	−0.108***	0.008	−0.108***	0.008	−0.109***	0.008	−0.108***	0.008
Age	0.050***	0.008	0.049***	0.008	0.048***	0.008	0.048***	0.008
Education	0.157***	0.008	0.153***	0.008	0.153***	0.008	0.153***	0.008
CTQ-R	−0.102***	0.008	−0.182***	0.012	−0.201***	0.013	−0.199***	0.013
CTQ-R^2^			0.041***	0.005	0.092***	0.013	0.091***	0.013
CTQ-R^3^					−0.011***	0.002	−0.011***	0.002
CTQ-R^4^							0.0003	0.001
*df*	14,618		14,617		14,616		14,615	
*AIC*	40,681		40,611		40,594		40,596	
*Log-Lik*	−20,334		−20,298		−20,289		−20,289	
*R*^2^	0.0559		0.0606		0.0618		0.0618	
*F1*	-		72.19 (*P <*.001)		45.71 (*P <*.001)		30.49 (*P <*.001)	
*F2*	-		72.19 (*P <*.001)		19.23 (*P <*.001)		0.06 (*P*= .811)	

*Note:*

* *P*< .05, **P <.01, ^***^P< .001. F1 tested the significance of the incremental explained variance compared to the linear model. F2 tested the significance of the incremental explained variance compared to the previous model.

**Table 2.** Top association findings (*P* < 5 × 10^-8^) in the GWAS analysis of future orientation and interaction with early adversity

SNP	CHR	BP	A1	A2	NMISS1	BETA1	SE1	NMISS2	BETA2	SE2	Z_G × E	P_G × E
rs4076431	1	37000466	G	A	3303	0.09	0.03	3201	−0.13	0.03	5.51	3.67 × e^−08^
rs10752589	1	37001923	T	C	3303	0.10	0.03	3201	−0.12	0.03	5.47	4.62 × e^−08^
rs9660229	1	37004527	A	G	3303	0.10	0.03	3201	−0.13	0.03	5.52	3.31 × e^−08^
rs4498771	1	37005270	A	G	3303	0.10	0.03	3201	−0.13	0.03	5.54	2.97 × e^−08^

*Note*: BETA1, regression coefficient in CTQ-R controls; SE1, standard error of coefficient in CTQ-R controls; NMISS1, number of non-missing genotypes in CTQ-R controls; BETA2, regression coefficient in CTQ-R cases; SE2, standard error of coefficient in CTQ-R cases; NMISS2, number of non-missing genotypes in CTQ-R cases; *Z, Z* score, test for interaction; *P*, asymptotic *P*-value for interaction test.

To test the robustness of this curvilinear relationship in different population subgroups, we examined the interaction between demographics (sex, age and education) and CTQ-R. Results showed that age had a significant interaction effect with the linear term of CTQ-R, *β* = 0.032, 95% CI for *B* = [0.015, 0.049], *t* = 3.68, *P* < .001. The negative relationship between CTQ-R and future orientation became weaker as age increased. We did not detect any significant interaction between sex or education with any polynomial of CTQ-R. The linear, quadratic and cubic terms of CTQ-R remained significant after the inclusion of the interaction effect of age, supporting the generality of the curvilinear relationship between CTQ-R and future orientation in heterogeneous populations.

### GWAS on future orientation

We conducted a GWAS study testing the effect of the childhood maltreatment status and SNP allele on future orientation (dependent variable) of ∼6.8 million variants using up to 14,675 WeGene Biobank participants. The top-hit SNP rs4498771 and its linked SNPs located in the intergenic area around CSF3R were discovered. We used the FUMA platform (Watanabe *et al.*, 2017) to confirm that CSF3R is the nearest gene from rs4498771, which is located ∼50kb away from the loci.

**Figure 2.**
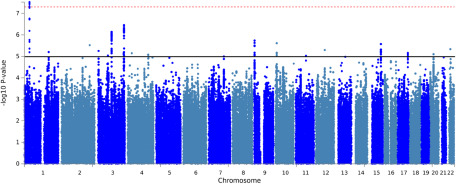
Manhattan plot of GWAS associations.

*Note*: The x-axis is chromosomal position and y-axis is the -log10 *P*-value of associations with future orientation effect. Significant (*P* = 5 × 10^-8^) and suggestive (*P* = 1 × 10^-5^) genome-wide threshold are shown by red and black lines.

Manhattan and QQ plots are shown in Fig. 2 and Supplementary Figure S1. There was no evidence of GWAS inflation (*λ* = 1.01). GWAS analysis identified a significant G × E effect (*P*< .001) at an intergenic locus on chromosome 1 (top-hit SNP, rs4498771, *P* = 2.97 × 10^−8^, closest gene: CSF3R). The regional visualization plot of rs4498771 is shown in Supplementary Figure S2.

To clarify the interaction effect between gene and childhood maltreatment on shaping individuals’ future orientation, a gene × stress interaction analysis of single locus was performed. Taking the top-hit SNP rs4498771 detected in the previous GWAS analysis as the candidate gene, childhood maltreatment as the environmental stress variable, and future orientation as the dependent variable, regression models were estimated to examine the main and interaction effects of the candidate gene.

### Candidate gene analyses: G × CTQ-R interaction on future orientation

We used regression analyses to examine the interaction between the frequency of childhood maltreatment and genetic variation at CSF3R. The cubic polynomial model was used as the base model, and the G × childhood maltreatment interactions were used as fixed-effect factors for predicting individual differences in future orientation scores. Sex, age and education were included as covariates in the models. Their interactions with genes and with childhood maltreatment were also tested to control for the effects these variables may have on the G × E interaction (Keller, 2014). Only age had a significant interaction with the linear term of early adversity, so this interaction term was included in the final model. Table 4S shows the results of the final regression model.

The main effects of genotype were significant (*β* = 0.026, 95% CI = [0.003, 0.049], *t* = 2.23, *P* = .03), as well as the main effect of all polynomials of CTQ-R (*β*_1_ = −0.200, 95% CI = [−0.225, −0.174], *t* = 15.23, *P* < .001; *β*_2_ = 0.238, 95% CI = [0.068, 0.118], *t* = 7.34, *P* < .001; *β*_3_ = 0.122, 95% CI = [−0.015, −0.006], *t* = 4.32, *P* <.001). The interaction effects of genotype with all polynomials of CTQ-R were also significant (*β*_1_ = 0.056, 95% CI = [0.031, 0.082], *t* = 4.36, *P* <.001; *β*_2_ = −0.040, 95% CI = [−0.065, −0.015], *t* = 3.16, *P*<.001; *β*_3_ = 0.006, 95% CI = [0.001, 0.011], *t* = 2.51, *P* = .012).

**Figure 3.**
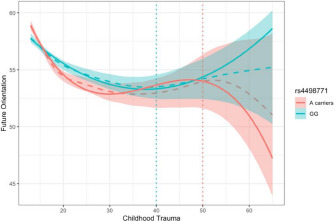
Gene × CTQ-R interaction on FCI.

*Note*: CTQ: childhood trauma questionnaire; FCI: future-orientated coping inventory. The dashed line represents the loess smoothing of the raw data, the solid line represents the quadratic (green) or cubic (red) polynomial regression as an approximation, and the dotted vertical lines mark the critical turning points of each curve.

Simple slope analysis as a follow-up to the interaction effects examined the differential curvilinear relationships between childhood maltreatment and future orientation within each genotype group. Specifically, the effects of linear, quadratic and cubic terms of CTQ-R on future orientation were examined in each genotype of rs4498771 (A carries and GG). The interaction effect is depicted in Fig. 3.

The linear and quadratic terms of CTQ-R were significant in both genotype groups, for A carriers, *β*_1_ = −0.253, 95% CI = [−0.289, −0.218], *t =*13.94, *P* < .001, *β*_2_ = 0.133, 95% CI = [0.098, 0.167], *t* = 7.52, *P* < .001, for GG, *β*_1_ = −0.145, 95% CI = [−0.182, −0.108], *t =*7.64, *P* < .001, *β*_2_ = 0.052, 95% CI = [0.016, 0.088], *t* = 2.86, *P*< .01; however, the cubic terms were only significant in the A carriers, for A carriers, *β*_3_= − 0.017, 95% CI = [−0.024, −0.010], *t =*4.80, *P* < .001, for GG, *β*_3_ = −0.004, 95% CI = [−0.011, 0.002], *t =*1.24, *P* = .214, indicating that individuals in the genotype group of A carriers showed a cubic association between childhood maltreatment and future orientation; that is, after experiencing frequent childhood maltreatment (in Fig. 3, at the turning point of the red line, CTQ-R total score = 50, CTQ-R item mean = 4 ‘often true’), future orientation exhibited a sharp decline. Contrastingly, the GG genotype group showed a quadratic association (U-shape) between childhood maltreatment and future orientation. Expressly, after experiencing some childhood maltreatment (in Fig. 3, at the turning point of the green line, CTQ-R total score = 40, CTQ-R item mean = 3 ‘sometimes true’), future orientation exhibited a rebound rather than a sustained decline.

Split-half cross-validation was performed to test the robustness of the findings. The results replicated the above pattern in both halves of the data; the more resilient genotype GG exhibited a quadratic U-curve, whereas the more vulnerable genotype A carriers exhibited a cubic curve decline. These curves in both halves of the data can be found in the Supplementary Figures.

## Discussion

The present study showed consistent evidence for gene × childhood maltreatment interaction in predicting future orientation. We demonstrated the effect of an interaction of the rs4498771 located in the intergenic area around CSF3R with childhood maltreatment on future orientation in a large nationwide sample involving Chinese adults.

In the GWAS study, CSF3R was a moderator between childhood maltreatment and future orientation. CSF3R is a granulocyte colony-stimulating factor receptor mRNA, a gene that is closely related to the chronic psychological stress response (Kawai *et al.*, 2007; Le-Niculescu *et al.*, 2011; Morita *et al.*, 2005). Psychological stress activates the HPA axis, sympathetic nervous system and immune system. These systems interact to affect the stress response (Connor and Leonard, 1998; Raison and Miller, 2003). In addition to the corticotrophin-releasing hormone, adrenocorticotropic hormone and glucocorticoids, stress stimulates production of cytokines and modifies inflammatory and immune responses. This physiological process is linked to peripheral leukocytes, which produce various cytokines and proinflammatory cytokines, particularly gp130 family members, and directly activates the HPA axis (Arzt, 2001). Leukocytes express receptors for stress mediators, such as hormones, neurotransmitters, growth factors and cytokines. *CSF3R* is a gene that affects the expression of the cytokine receptor. Different CSF3R genotypes may have different levels of expression, and therefore result in different cytokine production and stress responses. Therefore, studying CSF3R genotypes may be useful to objectively assess psychological stress responses. CSF3R is a candidate genetic factor contributing to stress (Kawai *et al.*, 2007; Le-Niculescu *et al.*, 2011; Morita *et al.*, 2005); however, there is a paucity of research on this gene as an indicator of individual differences and its involvement in the pathway from the stressful experience to the development of future orientation.

Theoretical model has been developed to conceptualize ‘stress inoculation’, which assumes that individuals with effective coping may keep or even increase their psychological strength after experiencing a moderate level of adversity (Dienstbier, 1989; Garmezy, 2016; Meichenbaum, 2017). This study put the stress inoculation theory into practice on childhood maltreatment, and the results challenged the previously assumed inverted U-shaped curve: instead of a universal curve for all, a positive U-curve best fits the data for some genotypes and a cubic curve best fits others. All participants demonstrated a decrease in future orientation when early trauma increased from none to moderate, but after a certain threshold, individuals with particular genotypes stopped declining and even showed a rebound (a positive U-curve), while individuals with other genotypes showed an accelerated decline (a cubic curve). Notably the environmental stress variable we chose was childhood maltreatment, which has a higher level of stress than the moderate stress described in the stress inoculation model. Therefore, a possible explanation concerning the failure of the inverted U-curve may be that childhood maltreatment is more damaging than ordinary stressful events, and thus any exposure to this type of event would likely have negative consequences. However, when accumulated stress reaches a certain threshold, some people may learn from it or are motived to develop more positive resources, thereby achieving stress-related growth, while others may feel hopeless and give up, thus losing the opportunity to grow.

Our findings extended the results of previous G × E studies of the CSF3R polymorphism and found a main effect of CSF3R and confirmed that individuals with certain genotypes of CSF3R tended to be more resilient and continue developing future orientation despite childhood adversity, while other genotypes did not. This is consistent with the suggestions of Drury *et al.* (2010), who implied that the beneficial effect of different alleles varies in different environmental settings and different developmental time points (Drury *et al.*, 2010).

Our results did not support an optimal stress exposure dose that fosters better psychological resources, but rather a threshold dose after which a bifurcation emerges that distinguishes between the stress-related growth path and the post-stress deterioration path. Genetic factors played a role in which path is taken. Stress-related growth is more likely to occur in some genotypes than others. While it is relieving that some people may gain growth after traumatic events, we should be aware that others may be severely impaired by traumatic experiences, as evidenced by a dramatic decrease in future orientation after the threshold dose. These individuals may need more attention and support after experiencing traumatic events, whereas the potential to thrive after extreme adversity for some individuals cannot be underestimated.

One may argue that the use of subjective trauma measures has the limitation of retrospective self-reporting, and the self-recall bias in the CTQ may limit the representativeness of the measure. However, the development of psychopathology is determined by subjective rather than objective experiences of childhood abuse. Danese and Widom (2020) examined a unique cohort of 1196 children with both objective, court-recorded evidence of abuse and their subjective reports of their childhood abuse in adulthood. A history of maltreatment and an extensive psychiatric evaluation were conducted. They found that the risk of psychopathology associated with objective indicators was minimal, even in cases of severe child abuse confirmed by court records. Contrastingly, the risk of psychopathology associated with subjective reports was high, and these findings have implications for how we study the neurobiological impact of child maltreatment.

The greater relevance of subjective measures of trauma compared to objective measures can be attributed to several factors: First, the impact of trauma on an individual’s psychological well-being is influenced by their subjective perception and interpretation of the event. Two people may experience the same objective traumatic event but have vastly different psychological responses based on their unique perspectives, coping mechanisms and personal history. Traumatic events can have different meanings for different individuals. Subjective trauma measures recognize that the personal meaning and significance of an event can impact psychological well-being and the risk of developing psychopathology (Nelson *et al.*, 2020). Second, subjective trauma measures consider emotional reactions to events, which are crucial in understanding the severity of psychological distress. Emotional reactions to trauma, such as fear, helplessness or horror, can play a significant role in the development of psychopathology (Noll, 2021). Third, subjective measures consider an individual’s resilience and vulnerability factors, which influence how they process and recover from a traumatic event (Gee, 2021). These factors may include personal characteristics, support systems and coping strategies that can affect the likelihood of developing psychopathology.

The main contribution of our study to the literature is that we combined the stress inoculation theory with genetic individual differences and proposed the threshold model of stress-related growth, in which not only stress exposure levels but also individual differences, such as genotypes, are involved in stress-related growth. The development of this model enriched the person–environment interaction theory (Fig. 1). Specifically, when considering negative outcomes such as psychological disorders, the diathesis–stress theory (Monroe and Simons, 1991) explains the interaction process between a person and a negative environment, while the differential susceptibility theory (Belsky and Pluess, 2009) explains the interaction between a person with both a negative and positive environment. Then, the vantage sensitivity hypothesis (Pluess and Belsky, 2013) starts to address positive outcomes after positive environmental exposure. The threshold model of stress-related growth advanced in this study tackles an aversive situation when positive outcomes occur after negative environmental input, and therefore contributes to the completeness of the person–environment interaction theory. Moreover, the threshold model provides a nuanced description on the shape of the relationship and its promoting factors. Genotype may modulate risk and resilience at an individual level (Feder *et al.*, 2009; Rutten *et al.*, 2013); our hypothesis provides a genetic basis for psychological growth after stress. It implies that genetic composition, such as CSF3R, may explain, in part, why only some individuals develop positive psychological resources (i.e., future orientation) after adversity, while others do not (Phoolka, 2012).

The second contribution of this study is that it provides evidence using both GWAS and G × E interactions for a specific gene. Prior reports have indicated difficulty in replication and small sample sizes in G × E studies (Dick *et al.*, 2015), and have suggested the need for conducting GWAS as an unbiased approach for discovery. Moore explained the advantages of G × E protocol in psychological research (Moore, 2017). Accordingly, many meta-analytical studies have integrated the effect size from different G × E studies to identify significant effects in a relatively large sample (Kim-Cohen *et al.*, 2006; Manning *et al.*, 2011), in which the reliability and validity of G × E research with candidate genes were increased. Sometimes, researchers consider SNPs implied by GWARS/GWAS when conducting candidate gene studies (Rietschel *et al.*, 2010). Concurrently, however, researchers have noted concerns about candidate gene studies in this area. The low reproducibility of replicated studies alone may be owing to the choice of environmental variables. Different choices of environmental variables lead to inconsistency between different sources of stress exposure resulting in publication bias (Duncan and Keller, 2011; Munafò *et al.*, 2009). Therefore, cross-validation of results obtained in the same population and stress exposure can enhance the validity of the results. The present results combined the two approaches and cross-validated that the CSF3R interacted with early adversities to result in future orientation. Our study found that specific types of gene are associated with distinct neurobiological changes (i.e., HPA), which could suggest a need for more targeted research approaches. Further, if certain neurobiological changes are associated with resilience to the impact of maltreatment, this could influence the development of therapeutic interventions. For example, interventions might aim to promote the neurobiological markers of resilience or counteract those associated with vulnerability.

The third contribution of this study is that it explored the curvilinear relationship between childhood maltreatment and future orientation under *a prior* hypotheses, in a large nationwide sample with robust results, thereby resulting in a more precise relationship among gene, childhood maltreatment and future orientation as one important aspect of resilience. Usually linear models are more robust, but if the actual phenomena are not linear, then allowing nonlinear terms can bring us closer to the truth. Since curve models are more likely to be overfit, we used a large sample size with cross-validation to increase the reliability and generalizability of our results. We hope this attempt can encourage other researchers to consider curvilinear relationships in theoretical and statistical modelling.

The present study has several limitations. First, the results we obtained were based on the results of Chinese Han people. After comparing the minor allele frequencies of the four candidate genes in different populations, there are differences in different ethnic populations, which may limit the generalizability of our study (Table S1). Further investigation of shared and nonshared genetic characteristics is therefore warranted, which could facilitate future integration of genetic–psychological trait association studies. Further, future studies are needed to replicate our findings, with additional SNPs in more genes, and using even larger samples (Chabris *et al.*, 2015). Second, excluding sexual abuse from the CTQ may limit the representativeness of the measures. Future studies should consider a more comprehensive assessment of environmental variables. Third, although future orientation is an important indicator of resilience and research findings on it can inform our understanding of resilience, it is not fully equivalent to resilience. Future research could consider more resilience indicators and resources (e.g., optimism, hope, self-efficacy, etc.) to form a more valid resilience factor and further verify the stress-related growth hypothesis (Gan *et al.*, 2019; Ord *et al.*, 2020). Fourth, although we used sex, age and education as covariates in all analyses, other variables, including socioeconomic status, may affect future orientation and are important in terms of both resilience and vulnerability. Fifth, compared with the curvilinear effect of CTQ, the effect size of genotype is relatively small, yet robust. We randomly divided the entire dataset into two parts and consistently observed the replication of the cubic effect and the interaction of genotype in each subset of data. As a methodological simulation study by Ganzach (1997) revealed, failing to incorporate appropriate quadratic and product terms into the regression equation can lead to a misinterpretation of the true relationship. Therefore, the interaction effect of genotype should not be overlooked in our quest to uncover the unbiased relationship between CTQ and future orientation. In addition, the identification of CSF3R as an important moderator between stress and stress-related growth is preliminary as variables such as blood parameters (e.g., neutrophil count) (Dale and Link, 2009) and family history and current diagnosis of neuropathic pain (Zhang *et al.*, 2017) and Alzheimer’s disease (López-González *et al.*, 2015) may covary with CSF3R and help explain how CSF3R works. However, this large-scale study failed to consider these factors. Further research should focus on disentangling these complex relationships to elucidate how genetic and environmental factors jointly contribute to shaping future orientation (Rutter, 2006). Finally, neuroimaging studies are required to identify the neural pathways by which G × E interactions function.

## Supporting information

Gan et al. supplementary material Gan et al. supplementary material

## Data Availability

Data containing all other variables used in this study are publicly available, except for genetic data, which are contractually prohibited from sharing. https://osf.io/bmntf/?view_only=799d080d7559460288d4d8a7804458d8

## Supplementary material

The supplementary material for this article can be found at https://doi.org/10.1017/S2045796024000581.
