# Supplementary material for: Interactions of the CSF3R polymorphism and early stress on future orientation: evidence for the differential model of stress-related growth
Source: Epidemiol Psychiatr Sci. 2024 Oct 3;33:e44. doi: 10.1017/S2045796024000581 (PMC11464942; doi:10.1017/S2045796024000581)
Supplement: Gan et al. supplementary material [file S2045796024000581sup001.docx]

Supplementary Table 1

The Means and Standard Deviations of CTQ and FCI in Each CFS3R Groups

|  | | n | CTQ | | FCI | |
| --- | --- | --- | --- | --- | --- | --- |
|  |  |  | M | SD | M | SD |
| rs4076431 | G carriers | 7374 | 18.92 | 7.24 | 56.22 | 11.04 |
|  | AA | 7301 | 19.09 | 7.48 | 56.18 | 11.05 |
| rs10752589 | T carrier | 6664 | 18.97 | 7.29 | 56.18 | 11.07 |
|  | CC | 8011 | 19.03 | 7.42 | 56.22 | 11.03 |
| rs9660229 | A carrier | 7414 | 18.92 | 7.24 | 56.21 | 11.05 |
|  | GG | 7261 | 19.09 | 7.48 | 56.20 | 11.04 |
| rs4498771 | A carrier | 7409 | 18.92 | 7.24 | 56.20 | 11.05 |
|  | GG | 7266 | 19.09 | 7.48 | 56.20 | 11.04 |

*Note: CTQ means early adversity questionnaire; FCI means future-orientated coping inventory.*

Supplementary Table 2.

Top association findings (p<5 x 10-8) in the GWIS analysis of future orientation and interaction with early adversity

| SNP | CHR | BP | A1 | A2 | NMISS1 | BETA1 | SE1 | NMISS2 | BETA2 | SE2 | Z_G×E | P_G×E |
| --- | --- | --- | --- | --- | --- | --- | --- | --- | --- | --- | --- | --- |
| rs4076431 | 1 | 37000466 | G | A | 3303 | 0.09 | 0.03 | 3201 | -0.13 | 0.03 | 5.51 | 3.67×e^-08^ |
| rs10752589 | 1 | 37001923 | T | C | 3303 | 0.10 | 0.03 | 3201 | -0.12 | 0.03 | 5.47 | 4.62×e^-08^ |
| rs9660229 | 1 | 37004527 | A | G | 3303 | 0.10 | 0.03 | 3201 | -0.13 | 0.03 | 5.52 | 3.31×e^-08^ |
| rs4498771 | 1 | 37005270 | A | G | 3303 | 0.10 | 0.03 | 3201 | -0.13 | 0.03 | 5.54 | 2.97×e^-08^ |

*Note:*

*CTQ: total score of Childhood Trauma* *Questionnaire;*

*CTQ value ≤13 as controls vs. CTQ value ≥23 as cases.*

*BETA1, regression coefficient in* *CTQ controls; SE1, standard error of coefficient in CTQ controls;* *NMISS1, number of non-missing genotypes in CTQ controls; BETA2, regression coefficient in CTQ* *cases; SE2, standard error of coefficient in CTQ cases; NMISS2, number of non-missing genotypes in CTQ cases; Z, Z score, test for interaction; P, asymptotic p-value for interaction test.*

Supplementary Table 3.

The moderating effect of genotype between future orientation and early adversity

|  | G=rs4076431 | | G=rs4498771 | | G=rs9660229 | | G=rs10751589 | |
| --- | --- | --- | --- | --- | --- | --- | --- | --- |
|  | B | SE | B | SE | B | SE | B | SE |
| CTQ | -0.199^***^ | 0.013 | -0.199^***^ | 0.013 | -0.199^***^ | 0.013 | -0.199^***^ | 0.013 |
| CTQ^2^ | 0.092^***^ | 0.013 | 0.092^***^ | 0.013 | 0.092^***^ | 0.013 | 0.091^***^ | 0.013 |
| CTQ^3^ | -0.011^***^ | 0.002 | -0.011^***^ | 0.002 | -0.011^***^ | 0.002 | -0.011^***^ | 0.002 |
| Genotype | 0.024^*^ | 0.012 | 0.026^*^ | 0.012 | 0.026^*^ | 0.012 | 0.025^*^ | 0.012 |
| CTQ x G | 0.057^***^ | 0.013 | 0.057^***^ | 0.013 | 0.056^***^ | 0.013 | 0.055^***^ | 0.013 |
| CTQ^2^ x G | -0.039^**^ | 0.013 | -0.041^**^ | 0.013 | -0.041^**^ | 0.013 | -0.037^**^ | 0.013 |
| CTQ^3^ x G | 0.006^*^ | 0.002 | 0.006^**^ | 0.002 | 0.006^**^ | 0.002 | 0.006^*^ | 0.002 |

*Note: ^*^p<.05, ^**^p<.01, ^***^p<.001. Gender, age and education were controlled in all models.*

CTQ: total score of Childhood Trauma Questionnaire.

Sup Table 4.

The moderating effect of genotype between future orientation and early adversity

|  | G=rs4498771 | |
| --- | --- | --- |
|  | B | SE |
| CTQ-R | -0.199^***^ | 0.013 |
| CTQ-R^2^ | 0.092^***^ | 0.013 |
| CTQ-R^3^ | -0.011^***^ | 0.002 |
| Genotype | 0.026^*^ | 0.012 |
| CTQ-R x G | 0.057^***^ | 0.013 |
| CTQ-R^2^ x G | -0.041^**^ | 0.013 |
| CTQ-R^3^ x G | 0.006^**^ | 0.002 |

*Note: ^*^p<.05, ^**^p<.01, ^***^p<.001. Gender, age and education were controlled in the current model.*


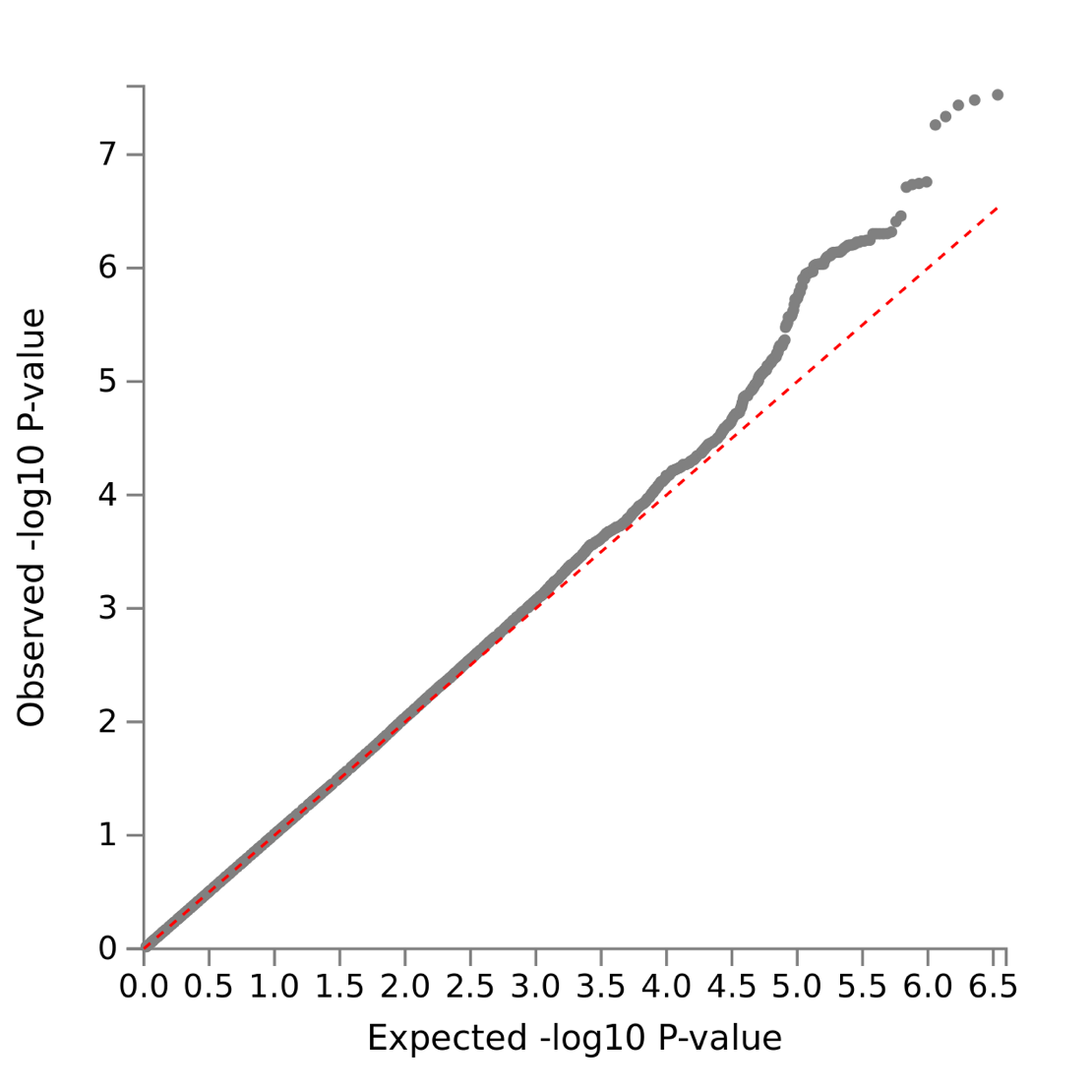


Supplementary Figure 1

Q–Q plots of the observed P-values

Supplementary Figure 2

The locus zoom plot for the associated region


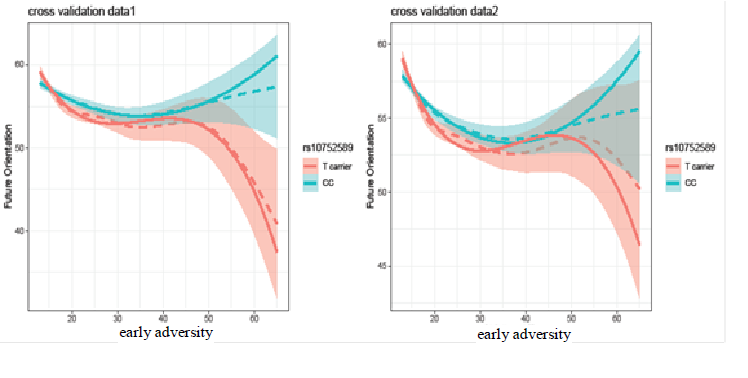


Supplementary Figure 3

An example of the different curvilinear relationships between CTQ and FCI in different genotypes using the cross validation data.
